# Protein import in mitochondria biogenesis: guided by targeting signals and sustained by dedicated chaperones

**DOI:** 10.1039/d1ra04497d

**Published:** 2021-10-01

**Authors:** Anna-Roza Dimogkioka, Jamie Lees, Erik Lacko, Kostas Tokatlidis

**Affiliations:** Institute of Molecular Cell and Systems Biology, College of Medical, Veterinary and Life Sciences, University of Glasgow University Avenue Glasgow G12 8QQ Scotland UK Kostas.Tokatlidis@glasgow.ac.uk

## Abstract

Mitochondria have a central role in cellular metabolism; they are responsible for the biosynthesis of amino acids, lipids, iron–sulphur clusters and regulate apoptosis. About 99% of mitochondrial proteins are encoded by nuclear genes, so the biogenesis of mitochondria heavily depends on protein import pathways into the organelle. An intricate system of well-studied import machinery facilitates the import of mitochondrial proteins. In addition, folding of the newly synthesized proteins takes place in a busy environment. A system of folding helper proteins, molecular chaperones and co-chaperones, are present to maintain proper conformation and thus avoid protein aggregation and premature damage. The components of the import machinery are well characterised, but the targeting signals and how they are recognised and decoded remains in some cases unclear. Here we provide some detail on the types of targeting signals involved in the protein import process. Furthermore, we discuss the very elaborate chaperone systems of the intermembrane space that are needed to overcome the particular challenges for the folding process in this compartment. The mechanisms that sustain productive folding in the face of aggregation and damage in mitochondria are critical components of the stress response and play an important role in cell homeostasis.

## Abstract 2

Mitochondria have a central role in cellular metabolism; they are responsible for the biosynthesis of amino acids, lipids, iron–sulphur clusters and regulate apoptosis.

## Graphical abstract

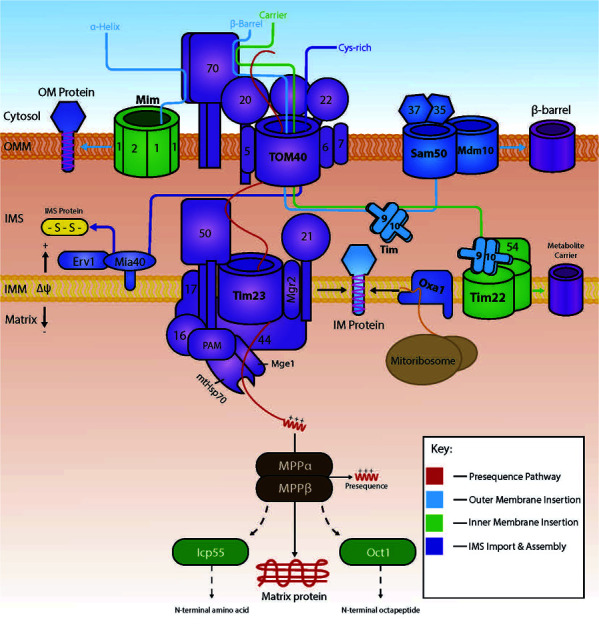

## 1. Introduction

Mitochondria are subcellular organelles that are critically important for cell physiology and development. Mitochondria have a central role in cellular metabolism; they are responsible for the biosynthesis of amino acids, lipids, iron–sulphur clusters, haem, and lipids. In addition, they are crucial in cellular signalling pathways and apoptosis.^1,2^ Moreover, mitochondria participate in a range of innate immunity pathways.^3^

Mitochondrial biogenesis requires the integration of two genomes, the nuclear and the mtDNA genome. Despite the plethora of mitochondrial functions, the mitochondrial genome only codes for a small set of proteins, namely 8 proteins in the yeast *S. cerevisiae* and 13 proteins in humans.^4^ These proteins are all core subunits of the electron transfer complexes located at the inner mitochondrial membrane. Approximately 99% of mitochondrial proteins are encoded by nuclear genes and are synthesized as protein precursors in the cytosol. Subsequently they are targeted to the surface of mitochondria and then sorted within their specific sub-mitochondrial compartment.^5^ The mitochondrial proteome contains about 1000 proteins in yeast and 1500 proteins in humans, fulfilling the mitochondrion's multiple functions.^1,2^ Proteomic studies have led to the functional classification of these proteins in yeast and more recently in human mitochondria. An inventory of a little over 1000 genes encoding the mammalian mitochondrial proteome, known as MitoCarta, was created in 2008.^4^ This was updated in 2015 to include a total of 1158 human or mouse genes leading to MitoCarta2.0.^6^ The latest MitoCarta release in 2020 includes 1136 genes with information on the submitochondrial localisation and the mitochondrial functional pathways they belong to.^7^

According to the inventory, the majority of the proteins are assigned to the matrix (46%) and the inner membrane (32%). 10% are annotated as outer membrane proteins whilst only 5% are designated as intermembrane space (IMS) proteins.^7^ Despite making up a small proportion of the mitochondrial proteome, proteins of the outer membrane and the IMS play a crucial role in the numerous functions of the mitochondrion. These include, apoptosis, phospholipid biosynthesis, haem biosynthesis and critical communications with the cytosol and other organelles. In addition, although the IMS is the smallest mitochondrial subcompartment, it encompasses a very wide range of protein import mechanisms. Protein import is pivotal for the mitochondrial intermembrane space (IMS) since all IMS proteins are encoded by nuclear genes.^2,8^

In this review we discuss the import pathways required to accommodate for the different types of proteins that are imported into mitochondria. Furthermore, we highlight the types and structural features of mitochondrial protein targeting signals that underpin the different import pathways. We then focus on the IMS, which is the only mitochondrial compartment housing a redox modification machinery integral to the import process. We discuss how this machinery works and how it facilitates a chaperone-assisted folding process in this compartment. Finally, we discuss the various chaperone systems in the IMS which are critical for sustaining an efficient folding environment in this compartment.

## 2. General protein import pathways into mitochondria

**Fig. 1:**
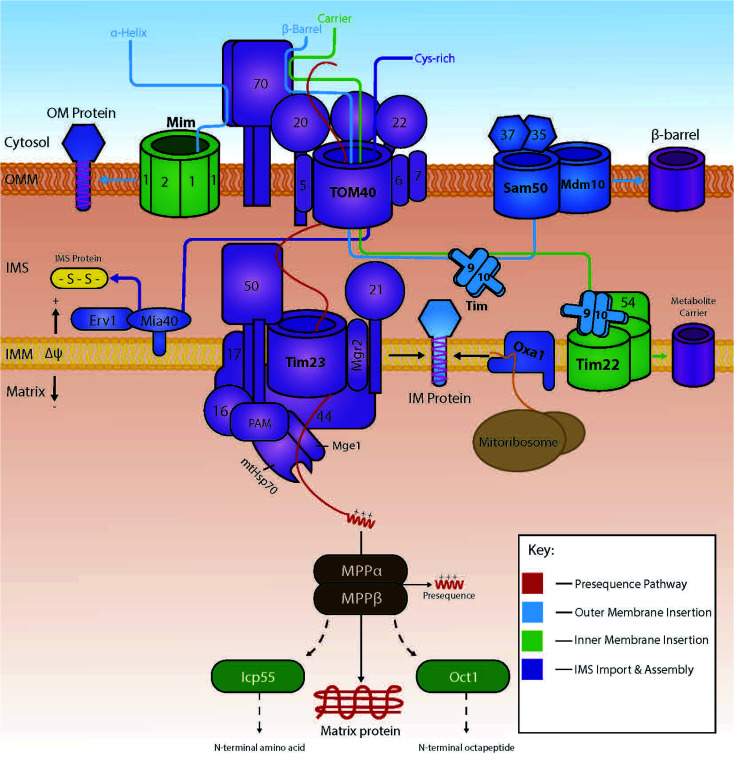
The various import pathways of preproteins into mitochondria. Proteins targeted to the mitochondrial matrix typically follow the presequence pathway (shown in red), where they are finally modified in the matrix by matrix processing proteins (MPP, ICP or Oct). Preproteins destined for the mitochondrial outer membrane are either integrated *via* the MIM machinery or by the SAM complex (depending on whether the protein is an α-helical protein or a β-barrel protein respectively, shown in light blue). Hydrophobic proteins destined for the inner membrane, are targeted and carried across the IMS by the Tim9/10 complex, and inserted by the Tim22 complex (shown in green). Proteins destined for the IMS are imported *via* TOM40 and modified into mature proteins by interactions with the oxidoreductase Mia40 (shown in dark blue).

Several import pathways are needed to accommodate the different types of proteins that are imported into mitochondria (Fig. 1). Each of the import pathways has specialised protein import components, many of which are encoded by genes that are essential for cell viability.^9^ The precursors undertaking a specific import pathway are guided by peptide targeting signals that are variable in length and their chemical properties and, in some cases, not very well understood. In the next paragraphs, we will summarise the main protein import pathways in terms of the components involved; the energetic requirements and the mechanism of interactions that ensure the efficiency of the protein import process which is key to mitochondria biogenesis.

### 2.1 The presequence pathway for import into the matrix

Cytosolically made proteins encoded by nuclear genes but targeted to mitochondria for their function usually require a system of mitochondrial-linked chaperones and a targeting sequences that determine the protein's endpoint within the mitochondria. Proteins destined for mitochondria typically are translocated as preproteins; preproteins typically contain an N-terminal amino acid presequence that usually forms an α-helix with positively charged residues on one side.^10^ This sequence is commonly referred to as the matrix-targeting signal (MTS), and will ultimately lead to the translocation of the protein into the mitochondrial matrix.^11^

The preprotein is carried within the cytosol in an unfolded state maintained by the cytosolic chaperones Hsp70 and Hsp90.^12^ Upon close proximity with the outer membrane of the mitochondria, the preprotein is typically recognised by the membrane-bound receptors Tom20 and Tom22, both of which form part of the main translocase of the outer membrane (TOM) complex, together with Tom40, the main pore-forming subunit of the TOM complex.^2^ In addition to Tom20, Tom70 is one of the first identified import receptors of the mitochondrial OM.^13^ Both Tom20 and Tom70 have their own specific preference for substrate, alongside the fact they are non-essential but have the ability to rescue each other's function.^14^ Tom40 acts as the main protein import channel and is encoded by an essential gene.^15^ Tom40 is endowed with the capacity to allow passage of almost all proteins that are translocated to mitochondria, and it contains a specific region for the binding of different types of preproteins. Also included within the TOM complex are the subunits Tom5, Tom6 and Tom7, all of which are encoded by non-essential genes (in contrast to Tom40 which is encoded by an essential gene) for the optimisation of the function of the complex.^16–18^ The matrix targeting signal binds to a groove of Tom20 that recognises its hydrophobic residues;^19^ recognition by Tom20 leads to the binding and translocation through the outer membrane *via* Tom40;^20^ the preprotein then interacts with the presequence translocase of the inner membrane known as TIM23. This interaction between the preprotein and TIM23 drives preprotein translocation across the inner membrane, in partnership with membrane potential (Δ*ψ*).^21^ Coupled to the TIM23 complex is the presequence translocase-associated motor (PAM), containing the mitochondrial version of heat shock protein 70 (mtHsp70). This motor promotes the unidirectional movement of preproteins into the mitochondrial matrix in an ATP-dependent manner.^22^ Following successful import, the positively charged presequence is cleaved by the mitochondrial-processing peptidase (MPP).^23,24^ Additional processing enzymes are then available to stabilize and fold the protein into its active form. Examples include: the removal of destabilizing amino acids by 55 kDa intermediate cleaving and octapeptidyl peptidases (Icp55 and Oct1 respectively);^25^ and correct folding *via* the Hsp60–Hsp10 chaperonin complex.^26^

### 2.2 Insertion into the inner membrane

If a protein is destined for a location other than the matrix, it will usually include either additional targeting information on top of the matrix targeting signal or an entirely different targeting signal. Some proteins that are destined for the IMS interact first with the inner membrane and are then released into the IMS. In this case, they contain a bipartite targeting signal made of a matrix-targeting sequence followed by a specific hydrophobic sorting signal. These proteins are initially targeted to the TIM23 complex following the matrix-targeting route. However, when in the TIM23 complex they get arrested by the small hydrophobic segment which functions as a stop-transfer signal (stop transfer pathway).^27^ Once stalled within the TIM23 complex, they are then released laterally into the inner membrane with the help of a small hydrophobic protein known as Mgr2.^28^

A separate route for insertion of inner membrane proteins exists for very hydrophobic proteins that are subunits of the electron transport complex of the inner membrane and are encoded by the mitochondrial DNA. These proteins are synthesised in the matrix and inserted into the inner membrane form the matrix side by the OXA insertase.^29–31^

A major part of inner membrane proteins are helical multispanning proteins that belong to the family of solute transporters of the inner membrane. These have typically six membrane spanning segments and are responsible for the transport of small metabolites across the inner mitochondrial membrane.^32^ The insertion of these proteins in the inner membrane is governed by the carrier pathway.^33^ In this case, the imported proteins do not contain a presequence, but instead internal peptide sequences found within the more hydrophobic areas of the transmembrane segments of the protein play the role of targeting signals. Proteins with internal signals are carried by Hsp70 and Hsp90 to the outer membrane where they are recognised by the receptor Tom70.^12^ Following their release from the cytosolic chaperones, proteins travel into the intermembrane space *via* the Tom40 channel, presumably in a looped formation.^34^ Small intermembrane space chaperones known as small TIM chaperones (Tim9 and Tim10 or Tim8 and Tim13) then bind – in a heterohexameric complex – to the protein to prevent any aggregation occurring.^34–37^ Here, the proteins are then guided by the chaperones to the translocase of the inner membrane TIM22 complex. The TIM22 complex then inserts the proteins in a process that depends on Δ*ψ*, thus producing a multi-spanning protein within the inner membrane.^38^ The insertion of Tim23 is like the carrier pathway as it also uses small TIM chaperones to allow transport across the intermembrane space, albeit with the non-essential chaperones Tim8 and Tim13.^39–41^ An example of a preprotein lacking an N-terminal presequence is the ADP/ATP carrier (AAC), as targeting information to the inner membrane is instead found within its three modules.^42^

### 2.3 Insertion into the outer membrane

The insertion of outer membrane proteins also involves the TOM complex, amongst other complexes found within the outer membrane. One such complex is known as the sorting and assembly machinery (SAM) complex and is used for the insertion of outer membrane β-barrel proteins.^43^ β-Barrel proteins are characteristic prokaryotic proteins. Interestingly, these proteins are also found within eukaryotic mitochondria and chloroplasts, making their lineage unique to the rest of the cell.^14^ There are three main subunits found within the SAM machinery: Sam50 (the main component), Sam35 and Sam37. Mitochondrial preproteins access this machinery by interaction and translocation into the intermembrane space *via* the TOM complex's Tom70 receptor and Tom40 channel. Here, the proteins interact with the small TIM chaperones, allowing for the integration of the precursor proteins into the SAM complex, where they are subsequently inserted into the outer membrane.^44^

Although β-barrel proteins are exclusively localised in the outer and not the inner mitochondrial membrane, the outer membrane also houses α-helical membrane proteins. These are integrated in the OM by a dedicated machinery, the mitochondrial import complex (MIM complex).^45^ Single-spanning proteins with an N-terminal anchor and multi-spanning membrane preproteins are able to bypass the Tom40 channel and be imported into the outer membrane directly *via* the MIM complex, usually in partnership with the Tom70 receptor.^46^ Proteins Mim1 and Mim2 are crucial for the normal function of the complex; complete absence of Mim2 has been shown to lead to the impairment of mitochondrial protein import, defects in mitochondrial morphology and problems in the assembly of the TOM complex.^47^

### 2.4 Import into the intermembrane space: chemical modification during import *via* an oxidation-coupled mechanism

All proteins destined for the intermembrane space are encoded by nuclear genes, and thus are synthesised and translocated from the cytosol. For these proteins to reach the intermembrane space, they can include one of two unique properties: (1) they can contain a bipartite sequence as previously discussed, or (2) they can be retained in the IMS by an interaction with the mitochondrial intermembrane space import and assembly protein 40 (Mia40).^48^ Bipartite sequences are targeting signals – much like matrix targeting signals – albeit with an added transmembrane-domain-like hydrophobic region following the MTS as discussed in the previous section. These preproteins following the stop transfer pathway are subject to a double cleavage (i) by the mitochondrial processing peptidase (MPP) to remove the MTS and (ii) by the specific intermembrane space protease (IMP) that removes the stop transfer hydrophobic domain –to discharge the mature protein into the intermembrane space. This pathway is fuelled solely by mitochondrial membrane potential, and does not require ATP-hydrolysis in contrast to the matrix targeting pathway where ATP hydrolysis in the matrix is needed.^11,49–51^

**Fig. 2:**
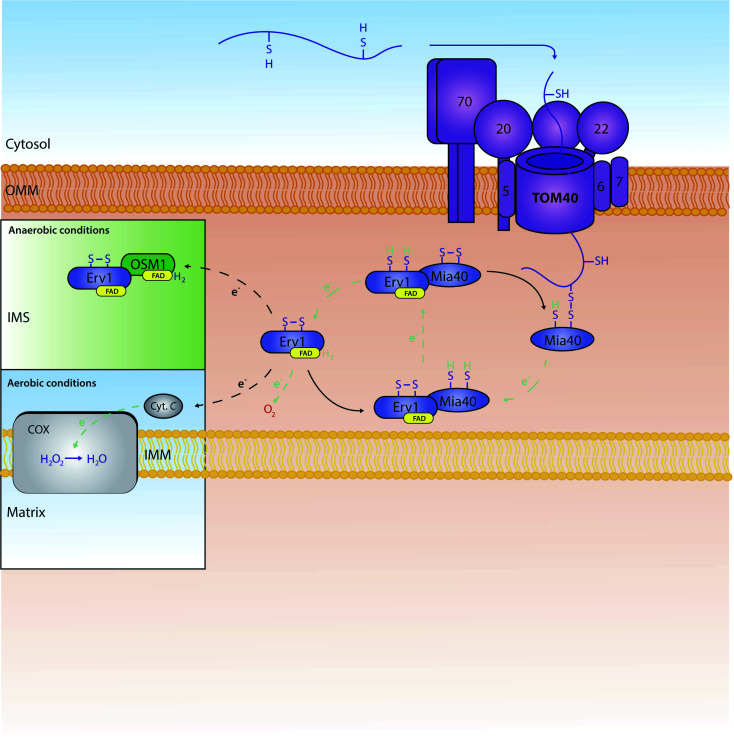
The role and recycling of the Mia40 oxidoreductase within the mitochondrial IMS. Proteins are actively oxidised by Mia40 leading to mature disulphide bond formation within the protein. Mia40 is subsequently reduced, and then re-oxidised by the FAD-bound Erv1. Erv1 is then re-oxidised by a number of proteins further down the chain. In aerobic conditions, Erv1 is recycled by O_2_ or cytochrome C (Cyt. C). In anaerobic conditions, Erv1 instead interacts with a fumarate reductase known as OSM1 which in turn transports electrons to fumarate.^69^ Oxidised Erv1 is then free to re-interact with Mia40 to complete the cycle and free up Mia40 to further interact with imported precursors.

A very substantial part of the intermembrane space proteins contain cysteine motifs – either CX_3_C or CX_9_C – which later become oxidised, forming strong disulphide bonds necessary for intermembrane space protein maturation. This discovery was based upon the presence of internal disulphides found within the TIM intermembrane space chaperones.^36,52^ This specific pathway is known as the MIA (mitochondrial protein import and assembly) pathway (Fig. 2), as preproteins involved in this pathway primarily interact with the oxidoreductase protein Mia40.^48^ In this interaction, Mia40 is bound to the inner membrane, leaving its C-terminus accessible to the intermembrane space; it is at this C-terminus where substrates interact with the oxidoreductase protein.^10^ Mia40 acts by ‘donating’ disulphides to premature intermembrane space proteins, thus leading to correct mature folding and entrapping within the intermembrane space. Previously stated CX_3_C/CX_9_C cysteine motifs interact with the specific substrate-binding cleft within the structure of Mia40. Here, the substrates then bind to what is known as a CPC motif, producing a disulphide intermediate between the substrate and Mia40.^53^ The specific substrate is then subsequently oxidised with correct disulphide formation leading to protein maturation and thus IMS protein activity. There are many examples of substrates of Mia40 that are vital to the normal function of mitochondria, such as the small TIM chaperones – Tim8, Tim9, Tim10, Tim12 and Tim13.^54^ Other examples include COX proteins – containing dual CX_9_C motifs – that are involved in the maintenance of the respiratory chain located in the mitochondrial inner membrane.^55,56^ Moreover, it seems that the import of other vital proteins (*e.g.* Mrp10 & Atp23 into the intermembrane space and Tim22 into the inner membrane) is dependent on their interactions with Mia40, albeit without the requirement of their cysteine motifs.^11^ Tim22 is a translocase that does not have any CX_*n*_C motifs but still relies on Mia40 for its correct import and insertion within the inner membrane.^10^ These interactions display the importance of this single oxidoreductase protein on the regulation and maintenance of mitochondrial intermembrane space proteins.

It was discovered that a sequence which is not an N-terminal sequence – and is common to many Mia40 substrates – exists within these substrates and is vital for the substrate's specific targeting capabilities to the mitochondrial intermembrane space. This intermembrane space targeting signal (ITS), consisting of 9 amino acids, is necessary for targeting of intermembrane-space-targeted preproteins to Mia40; the ITS functions by priming a cysteine residue within the substrate for effective docking with Mia40's active-site-resident CPC motif.^57^ Deletion of this ITS has shown to completely abolish the import of Mia40 substrates to the intermembrane space. The ITS functions by forming a helix with its vital hydrophobic and aromatic residues on the same side as this primed cysteine residue. Here, this region of the helix interacts with and induces the interaction of the substrate with the Mia40 oxidoreductase, ultimately leading to further interaction through disulphide bond formation.^57,58^

When Mia40 donates its disulphide to its substrates it stays reduced. To function again it needs to be recycled back to its oxidised state. This re-oxidation is ensured by a second component of the MIA pathway: a flavin adenine dinucleotide (FAD)-linked sulfhydryl oxidase known as Erv1 (Essential for respiration and viability 1).^59^ Erv1 is a Mia40 substrate with a structure unlike any other Mia40 substrate, containing three specific cysteine pairs that are highly conserved between species (residues C30/C33, C130/C133 and C159/C176);^11,56,59,60^ Erv1 is unable to oxidise proteins independently, although it can form a higher ternary complex with Mia40 to achieve this effect.^61^ Specifically, the first pair of cysteines are involved in the specific interaction of Erv1 with Mia40.^62,63^ The third pair of cysteines' disulphide bond has a more structural role; interestingly, this same disulphide is recognised by the Mia40 oxidoreductase during the import of Erv1 into the intermembrane space,^64^ with final folding and maturation of Erv1 occurring with FAD binding.^59^ Erv1 is a crucial component of the MIA oxidative folding pathway and functions by removing electrons from Mia40 and relaying them to either molecular oxygen or cytochrome c,^65,66^ thereby producing either hydrogen peroxide (H_2_O_2_) or water (H_2_O) respectively; water is produced from cytochrome c's funnelling of electrons to oxygen linking thus this oxidative folding pathway to the ETC. Specifically, Erv1 functions by collecting electrons (through Mia40's N-terminal CX_2_C motif) in its own CX_2_C motif. Here, the electrons are transferred onto FAD, where they can then be relayed to either molecular oxygen or cytochrome c. Although oxygen is generally the final electron acceptor, if electrons are relayed to cytochrome c, alternative structures are known to be final electron acceptors – such as the cytochrome c heme lyase Ccp1.^67^ In essence, through this specific electron relay, Erv1 recycles Mia40 from a substrate-induced reduced state to a functional oxidised state allowing Mia40 to continue its role as a ‘disulphide donor’. We should note that in human mitochondria, the Mia40 homologue (Mia40 or CHCHD4) translocation to the intermembrane space is dependent on the presence of the human homologue of Erv1 (ALR) and an internal targeting signal; this is due to the lack of an N-terminal membrane anchor that is present in the *S. cerevisiae* homologue of Mia40.^68^ This further shows the importance of Erv1 to Mia40's general function within the IMS.

## 3. Types and structural features of protein targeting signals for import into mitochondria

Mitochondrial targeting signals can be considered as ‘post codes’ that are necessary and sufficient when added to proteins for their submitochondrial localisation.^70^ About two thirds of all mitochondrial proteins are synthesized in the cytosol with a cleavable presequence. Presequences are of variable length, usually 18–50 amino acid residues, although very short (<10 aa) and very long (up to 100 aa) ones have been observed.^71,72^ An important characteristic of mitochondrial presequences is the formation of an amphipathic a-helix that contains a positively charged face and a hydrophobic face.^73^ The elements of the amphipathic helix are specifically recognised by receptors and other import components during preprotein translocation by the import machinery.^74^ Although the components of the import machinery have been well characterised as mentioned in the first part of this review, the cytosolic targeting steps and the proteins involved in these are less well-understood. Specifically, it is still not clear for many mitochondrial proteins whether their targeting signal can be recognised by one or many dedicated targeting factors, the specificity of such targeting factors and their interplay with the outer membrane receptor subunits that decode the targeting signals.

The main entry gate of precursor proteins to mitochondria is the translocase of the outer membrane (TOM) complex. The TOM complex consists of multiple subunits; Tom20 has been identified as the general receptor for protein sequences while Tom70 may also have a role in signal recognition.^75^ Tom20 has the monumental task of recognising roughly up to 1000 mitochondrial proteins, from non-mitochondrial ones, and recognising at least four different classes of precursor proteins with distinct targeting signals following various import pathways in the organelle.^76,77^ The import machinery not only functions as recognitions stations for the mitochondrial targeting signals but also as channels and driving forces for the translocation of the preproteins (Table 1).^74^

**Table 1:** Yeast mitochondrial proteins with N-terminal cleavable presequences. The positively-charged amino acid residues are shown in bold, negatively-charged residues are shown in italic and hydrophobic residues are shown in regular font. The cleavage sites processed by the mitochondrial processing peptidase (MPP) are mostly found in position R-2 and highlighted in cyan. Sequences were acquired from the *Saccharomyces* Genome Database (SGD)/UniProt and analysed using the MitoFates prediction tool for mitochondrial targeting sequences

Protein	Function	N-terminal presequence	MPP cleavage site
ACO1	Required for the tricarboxylic acid (TCA) cycle	…I**KR**PIV–	–RGLA…
HSP77	ATPase of the Hsp70 family, involved in protein translocation and folding	…AA**K**NILN**R**SS…	…RLQS…
TRX3	Thioredoxin required to maintain redox homeostasis in the cell	…FY**K**PVM**R**MAV**R**PL**K**SI–	–RFQS…
TUF1	Mitochondrial translation elongation factor	MSALLP**R**LLT**R**…	…RTFS…
XDJ1	Chaperone facilitating mitochondrial protein import; associated to Ydj1p	…*D***R**G*D***R**LY*D*VL…	…RKLA…
MXR2	Methionine-R-sulfoxide reductase, involved in the response to oxidative stress	…LG**KR**ICQ*E*AVT…	…RSGK…
SOD2	Manganese superoxide dismutase that protects cells against oxygen toxicity	…FA**K**TAAANLT**K**…	…RRTKV…

### 3.1 Matrix targeting signals

**Fig. 3:**
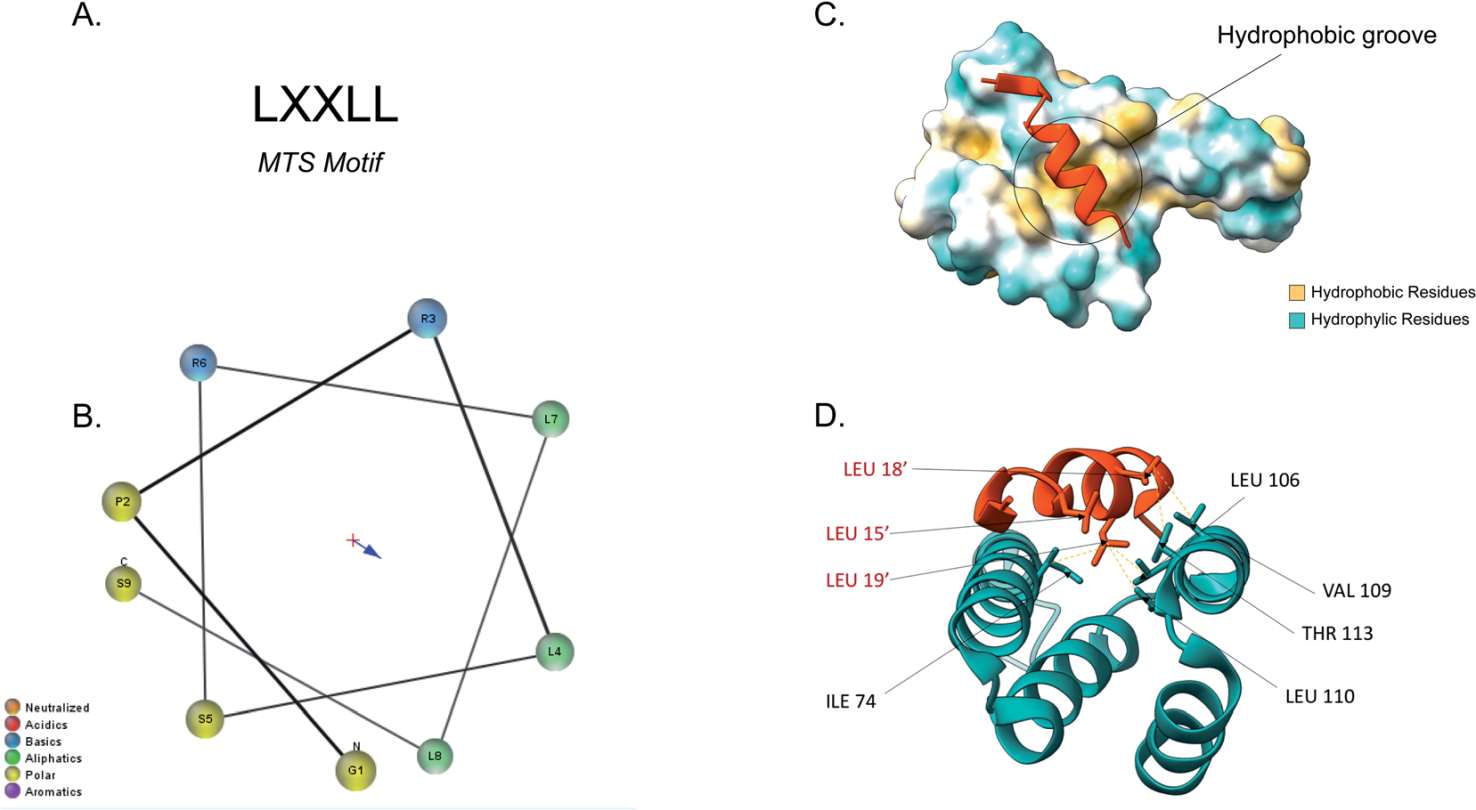
Tom20 interaction with the N-terminal presequence of aldehyde dehydrogenase (ALDH). (A) Matrix targeting sequence motif: L stands for leucine while X can be any amino acid. (B) Helical wheel projection of the presequence peptide amphipathic helix. The blue arrow points to the hydrophobic side of the helix. (C) Interaction of the ALDH presequence (orange) with the hydrophobic shallow groove of the helix-turn-helix domains of Tom20. The hydrophobic surface of Tom20 is indicated in yellow while the hydrophilic surface is light blue. (D) Contact residues of ALDH (orange) at the Tom20 interface. ALDH residues are represented in orange. Yellow dashes indicated contact between two atoms (<4.0 Å) (PDB ID: 2V1S).

The majority of the matrix proteins contain N-terminal cleavable mitochondrial sequences (MTS, Fig. 3). The MTS is 10–70 amino acids long, it is positively charged with a tendency to form an amphipathic α-helix (Fig. 3B).^71^ A comparative analysis of the N-proteome of mitochondria from mouse, human and yeast revealed that although the N-terminus sequence is poorly conserved, properties such as the length, charge and cleavage site are well conserved.^78^ These presequences are usually removed in the matrix, after import, by the matrix processing peptidase (MPP). MPP cleavage typically occurs at two amino acids C-terminal to an arginine (R-2).^24,71^

Tom20 recognises the hydrophobic side of the α-helix while Tom22 is presumed to recognise the basic residues of the presequences and thus complement the receptor function of Tom20.^19,79^ Previous analyses into the MTS consensus motif (Fig. 3A) revealed the following motif: LSRLL where L represents leucine (hydrophobic residue) and R is arginine (positively charged residue). Although this a typical motif, Tom20 has been shown to recognise a variety of this motif in other presequences such as the LRRAY of the mitochondrial heat shock protein 60 (Hsp60). Nevertheless, the three lysine residues appear to be the most important for preprotein recognition.^76^ Tom20 consists of two helix-turn-helix domains, that create a shallow groove that acts as the binding site for the presequence; the hydrophobic residues are indicated in yellow while the hydrophilic ones are presented in cyan (Fig. 3C). As seen in Fig. 3D, the three leucine residues (orange) of the presequence interact with residues in the groove (cyan). Leu15′ and Leu19′ are held in a hydrophobic created by Ile74, Leu106 and Leu110 side chains of Tom20. Leu18′ on the other hand, makes contact with the Val109 and Thr113 residues of the translocase.^76^

After recognition the presequence transverses the TOM complex by consecutively binding a number of binding presequence-binding sites spanning the channel formed by the β-barrel protein.^80,81^ These binding sites are arranged by in order of increasing affinity for the presequence, with the high-affinity binding site found at the IMS side of the channel.^82–85^ Next, the presequence has to interact with the translocation machinery of the inner membrane. The TIM23 channel consists of Tim23, Tim50 and Tim17; Tim23 and Tim17 are structurally similar and form the channel *via* which presequences are translocated.^86–88^ Tim50 was later discovered to have an important role in the transfer of proteins from the TOM to the TIM23 channel.^89–91^ Additionally, Tim50 regulates the conformation of the Tim23 channel by closing the channel in the absence of the presequence in order to prevent ion leakage. When the presequence binds Tom50, it subsequently leads to a conformational change in Tim23 that causes the channel to open.^77^ However, the two membranes are separated by the intermembrane space which is an aqueous sub-compartment. As a result, protein translocation needs to happen at the contact sites where the outer membrane and inner membrane are in close proximity. Once the presequence is translocated *via* the TOM complex, its N-terminus reaches the TIM23 complex of the inner membrane where a translocation intermediate is formed.^92–94^ The presequence is now threaded through both the TOM and TIM23 complex. This intermediate happens due to the lack of membrane potential in the outer membrane rendering the TOM complex a passive pore, incapable of completing the translocation of the mitochondrial preprotein. As a result, TIM23 provides a critical driving force for the translocation of the N-terminal presequences. TIM23 requires membrane potential and the activity of the matrix ATP-dependent translocation motor^14,74,95^ chaperone heat shock protein 70 (Hsp70) to drive the precursor protein across the inner membrane and into the matrix.^80,89,94,96^

However, the protein is not functional yet and reaches its mature form after the presequence gets cleaved by the MPP. MPP is a soluble matrix protein that processes the bulk of N-terminal presequences.^71,97^ It is a heterodimer consisting of two subunits: Mas1 which is the active site and Mas2 which contains a glycine-rich loop which plays an important role in presequence recognition.^98,99^ Although there is not a conserved cleavage site motif, most cleavage sites have an arginine residue situated two amino acids before the C-terminus (R-2, RXX) of the presequence.^71^ Another element that helps MPP recognise presequences for cleavage is an aromatic residue, such as phenylalanine or tyrosine, at position +1.^97,100^ MPP is not the only peptidase that can cleave N-terminal presequences as recently the human insulin-degrading enzyme (IDE) and its yeast homologue Ste23 have been linked to MTS cleavage and clearance.^101^ Although IDE is mainly found in the cytosol, an isoform resulting from the alternative translation of IDE localises to mitochondria and is believed to have an MPP-like function.^102^ Ste23 interacts with Cym1, an established peptide-degrading enzyme in the matrix to degrade presequence peptides and promote protein maturation.^101^ Presequence cleavage is not only important for protein maturation but also for cell viability. If MPP is impaired the preproteins will not mature and thus the accumulation of unfolded proteins might lead to aggregation in mitochondria.^101,103^

### 3.2 Inner membrane targeting signals

Most inner membrane proteins belong to a family of solute transport proteins (also called metabolite carrier proteins), such as the ADP/ATP carrier, and contain six α-helical transmembrane domains. Unlike the aforementioned cleavable presequences, carrier precursors contain internal targeting elements that remain part of the mature protein.^12,42^ The carrier protein precursors are guided primarily to the Tom70 receptor of the TOM complex by cytosolic chaperones. Nonetheless, there is growing evidence that Tom70 is not a selective receptor protein strictly involved in mitochondrial protein import and it functions as a versatile protein adaptor that facilitates the interaction of mitochondria with different proteins but also other organelles such as the ER.^104^ The carrier precursors are inserted into the Tom40 channel in a loop formation with both termini remaining exposed on the cytosolic side.^42,77^ Subsequently, chaperones are recruited in the IMS-side of Tom40 that facilitate the translocation of the hydrophobic precursors and protect them from aggregating or misfolding in the aqueous phase. These chaperones are the small Tims, Tim9–10 and Tim8–13, that we will discuss in detail in the next section. The transient binding of the importing carrier proteins to the small Tim chaperones is guided mainly by the transmembrane segments of the carrier proteins that function as the internal targeting signals.^36,40^ The presence of the entire sequence of the carrier proteins is presumably necessary to ensure the correct targeting of these proteins, since truncated versions of carrier proteins may be mistargeted to the matrix due to the presence of cryptic matrix targeting signals that would be rendered non-functional in the context of the entire protein and therefore allow proper targeting to the inner membrane.^105^ The small Tim chaperone complexes associate with the TIM22 complex of the inner membrane to aid the transfer of the precursor protein for final insertion into the membrane. The TIM22 complex consists of Tim18, Tim22 and Tim54, with Tim18 and Tim54 stabilising the complex. TIM22 also requires the membrane potential in order to facilitate the insertion of the transmembrane segments of the integral inner membrane proteins.^38,106–108^

### 3.3 Intermembrane space targeting signals

Although the IMS is the smallest mitochondrial subcompartment, it is vital for mitochondrial function. It constitutes a crucial buffer between the cytosol and matrix through the exchange of lipids, proteins, metals and other cofactors necessary for mitochondrial function, redox regulation and apoptosis.^10,71^ Additionally, there are numerous protein import pathways into the IMS perhaps indicative of its pleiotropic nature. One well-characterised type of targeting signal for the IMS is the bipartite presequence for a small number of IMS proteins. Additionally, for the majority of IMS proteins contain characteristic cysteine motifs^48,109^ and have their own unique targeting signal. The proteins with bipartite presequences contain an N-terminal mitochondrial targeting signal (MTS) as well as a hydrophobic sorting region. The N-terminal part of the presequence is similar to the cleavable mitochondrial targeting presequences, hence it is positively charged and forms an amphipathic helical structure. The positive domain of the bipartite sequence is recognised by the TOM complex receptors Tom20 and Tom22. This signal then allows the presequence to transverse the Tom40 channel. Once in the IMS side of the TOM complex (*trans* site), it is recognised by Tom50 that in turn feeds the presequence to TIM23.^74^ The latter region, known as the stop-transfer sequence, arrests translocation to the matrix inserted as the hydrophobic segment gets “stuck” in the Tim23 channel. The transmembrane domain is then laterally diffused and the presequence is anchored to the inner membrane with the N-terminal peptide facing the matrix. The MPP then cleaves the presequence to facilitate protein maturation.^101^ There is a secondary cleavage site between the hydrophobic segment and the mature protein facing the IMS. This motif is recognised and cleaved by inner membrane peptidase (IMP).^27,110^ The IMP is a heterodimer consisting of two different catalytic subunits; the Imp1 and Imp2 that are anchored to the inner membrane by their N-terminal domain. A third protein Som1 was shown to be involved in the IMP complex and was found to be essential for the proteolytic activity of the Imp1 subunit. The IMP complex cleaves the transmembrane segment leading to the release of the mature protein in the IMS.^49^

**Fig. 4:**
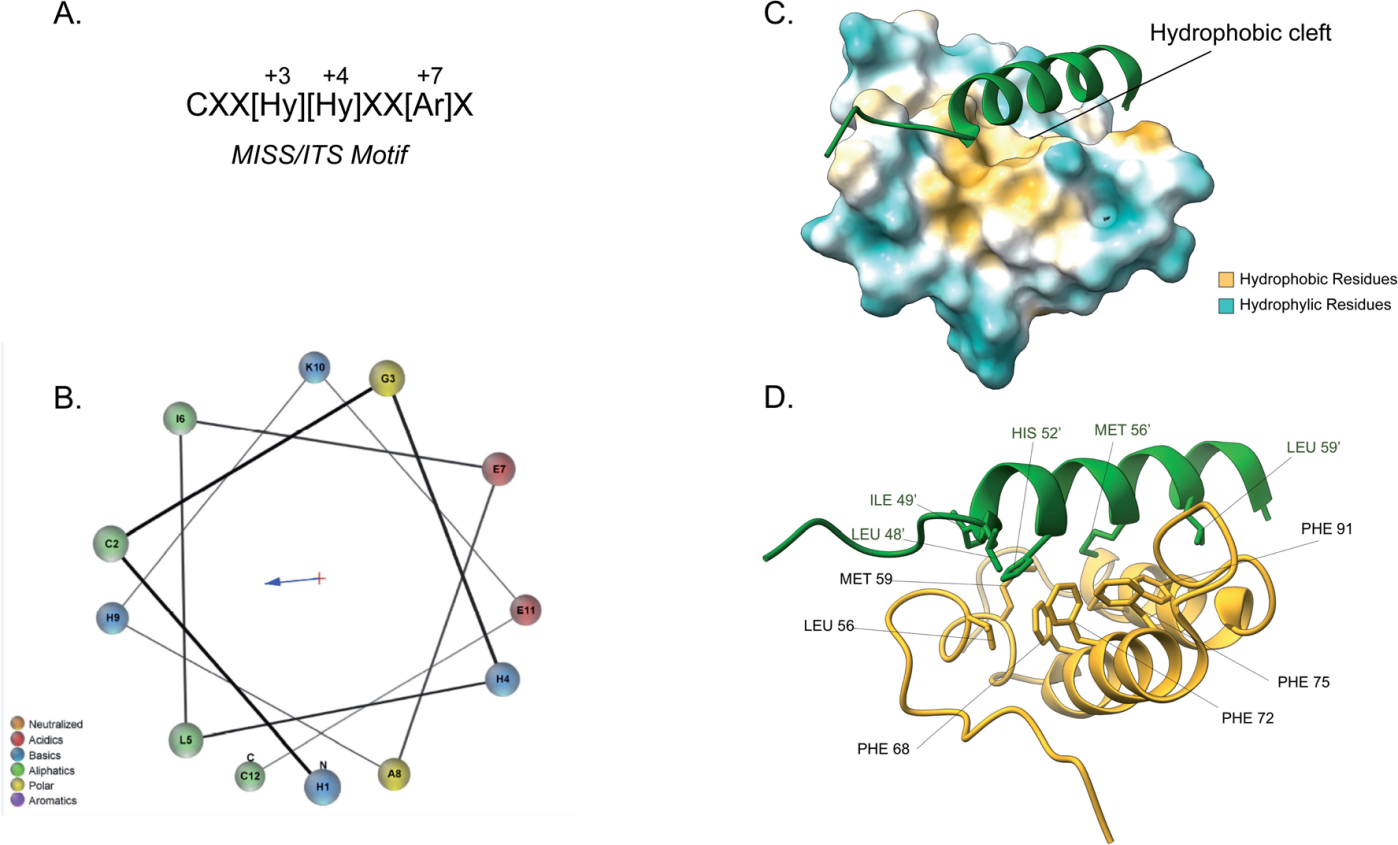
Mia40 interaction with the MISS/ITS peptide of Cox17. (A) Intermembrane space targeting sequence motif: L stands for leucine while X can be any amino acid. (B) Helical wheel projection of the presequence peptide amphipathic helix. The blue arrow points to the hydrophobic side of the helix. (C) Interaction of the Cox17 ITS/MISS (green) with the hydrophobic cleft of Mia40. The hydrophobic surface of Mia40 is indicated in yellow while the hydrophilic surface is light blue. (D) Contact residues of Cox17 (green) at the Tom20 interface. Cox residues are represented in green. Atom distance is <4.0 Å (PDB ID: 2L0Y).

The majority of intermembrane space proteins lack these bipartite presequences. Instead, they contain an internal, non-cleavable targeting signal with hydrophobic residues and characteristic cysteine motifs (CX_3_C and CX_9_C) that form intramolecular disulphide bonds as a consequence of their interaction with the MIA machinery. These targeting signals are termed mitochondrial IMS sorting signal (MISS) or the IMS targeting signal (ITS). The ITS peptide is usually nine amino acids-long and contains conserved residues that are important for both substrate recognition and folding of the substrate protein. The cysteine residues are usually separated by 3 or 9 amino acids, although some MIA substrates contain cysteines not organised in a specific motif. A comprehensive protein engineering and mutagenesis analysis has defined the consensus motif in the ITS signal, CXX[Hy][Hy]XX[Ar]X, where the hydrophobic residues in position −3 and −4 and an aromatic residue in position −7 are strictly conserved (Fig. 4A).^57^ The substrates of the Mia pathway are usually small proteins that remain reduced in the cytosol and are translocated to the IMS *via* the TOM channel. Once in the IMS, the ITS targeting signal is recognised by the oxidoreductase Mia40. The ITS forms an amphipathic α-helix with the hydrophobic face containing the conserved residues that are necessary for recognition and binding by Mia40 (Fig. 4B). Unlike the MTS peptide for targeting to the matrix, the charged residues of the ITS peptide are not functionally important.^57^ Recognition of the ITS by Mia40 occurs *via* site-specific hydrophobic interactions which allow a two-step disulphide bond formation event between the CPC site of Mia40 and the substrate's internal targeting signal (Fig. 4C and D).^11,53,57,111^ Folding of the substrate occurs *via* two consecutive induced folding events, each one coupled to one disulphide formation:

The first ITS helix folding is induced by the substrate cleft of Mia40, coupled to the formation of the intermolecular disulphide. The second helix is induced by the now folded ITS first helix, coupled to the intramolecular disulphide formation between the inner cysteines.

Covalent binding is thermodynamically essential to induce the α-helical folding of the Mia40 substrates and stabilise the protein–protein interactions. Once the disulphide bond between Mia40 and the substrate is formed, entropic contributions do not prevent the folding anymore and the formation of the α-helix is thermodynamically favoured. After substrate release, the Mia40 CPC motif remains reduced and gets reoxidised by Erv1 back to its functional state as an IMS receptor.^62,112,113^ The Mia40 has an expanded specificity by recognising substrates with unconventional cysteine motifs. For example, Mix23 displays a CX_14_C/CX_13_C and Erv1 is imported *via* a CXXC motif.

### 3.4 Atypical IMS targeting signals

In addition to the majority of IM proteins that contain an ITS targeting signal, there are proteins that have atypical targeting signal which guide them into the IMS.

#### The case of cytochrome proteins

Cytochrome b2 and cytochrome c1 follow the stop-transfer pathway, but their final localisation in the IMS depends additionally on a heme-binding domain (HBD) downstream of the stop-transfer signal. When their presequence is translocated across the outer membrane into the IMS, the HBD domain can fold independently of the presequence. As a result, the mature form of the HBD prevents the backward movement of the presequence and thus the presequence can only move forward into the IMS.^27,74,114^ The HBD domain also drives the import of cytochrome C into the IMS.^115,116^ Cytochrome C is one of the Cys containing proteins in the IMS that exceptionally does not depend on the MIA pathway for its import.^48^

#### Gpx3

Gpx3 is the major oxidative stress sensor in the cytosol. A ribosomal profiling study in *S. cerevisiae* provided evidence that under H_2_O_2_ stress conditions, ribosomal binding sites shifted upstream of the endogenous AUG codon leading to alternative translation.^117^ Gpx3 localises to the cytosol when translated through its canonical AUG start codon. However, under acute oxidative stress, an isoform of the protein is synthesized, with and 18-amino acid N-terminal peptide, from the translation of a non-AUG codon and is translocated to mitochondria. The N18 peptide is mostly hydrophobic and has positive charges but does not follow any of the conventional protein import pathways into the intermembrane space.^118^ Intriguingly, the Gpx3 without the N-terminal extension also gets targeted to mitochondria, albeit in lower yields than the N-extended form, suggesting that an as yet uncharacterised targeting signal must be present even in the mature part of the protein.^8^ Gpx3 is not the only protein that is dually localised in the IMS in addition to its cytosolic localisation. Other such proteins are the thioredoxin 1 (Trx1) and thioredoxin 2 (Trx2) and thioredoxin reductase Trr1, which are both targeted to the IMS by yet unknown targeting signals.^119^

### 3.5 Outer membrane targeting signals

The outer membrane possesses two types of integral membrane proteins: β-barrel and α-helical proteins. β-Barrel proteins are integrated to the outer membrane by multiple β-strands traversing the membrane while α-helical proteins are anchored to the membrane by one or more transmembrane α-helices.^2^ β-Barrel proteins make up two of the critical translocases of the protein import pathway (the Tom40 and Sam50 channel forming subunits of the TOM and SAM complex) and their function is therefore essential. They are synthesised in the cytosol like 99% of mitochondrial proteins but do not contain a cleavable targeting signal. Instead, the targeting signal of β-barrel proteins is internal to the protein and remains part of the mature protein. At first, the β-strands on the proteins were believed to be the targeting signal for these proteins but no specific motif was identified.^120^ However, it was later discovered that the actual targeting signal consists the most C-terminal β-strand called the β-signal;^121^ the properties of the β-signal have been further dissected to include high hydrophobicity and form a beta hairpin element that can extend to include two adjacent β strands that specifically bind the translocation machinery.^122^ The β-hairpin is recognised by Tom20 and subsequently translocated through the Tom40 channel. Upon translocation, TIM chaperones in the IMS bind to precursor in order to prevent aggregation in the aqueous sub-compartment. It is then transferred to the SAM complex, which is the central machinery for the β-protein biogenesis pathway. The SAM complex contains Sam50, a β-barrel protein itself, which is integrated in the outer membrane, as well as Sam35 and Sam37 that are peripheral membrane proteins exposed to the cytosol. Sam35 and Sam50 cooperate in the recognition of the β-hairpin motif and direct membrane insertion. The β-signal is then proposed to induce the opening of the lateral gate of Sam50 which leads to the lateral release of the protein to the outer membrane.^2,121^ Sam37 is understood to support the release of the β-barrel protein from the SAM complex to the OM but the exact mechanism of the release is not understood in great detail.^123^

The other group of OM proteins are α-helical OM proteins that are anchored to the membrane by one or more α-helices. There are no known examples of OM proteins that will have both β-barrel structure and α-helical transmembrane domains. They have various functions from protein transport to apoptosis. The biogenesis of these proteins is only understood in part. It appears that the positive charge and moderate hydrophobicity of their membrane anchor acts as a targeting signal.^124^ They are split into three main protein classes: signal-anchored, tail-anchored and polytopic outer-membrane proteins. Signal-anchored and tail-anchored proteins contain an α-helical transmembrane section at the N- and C-terminus, respectively. The transmembrane domain and its flanking regions function as both a membrane anchor and a targeting signal. The exact targeting signal for polytopic proteins is not known but it may be associated with the multiple transmembrane domains of the proteins. Tom70 is believed to be the receptor for such proteins but it is unclear if they pass through the Tom40 channel for their import.^125^ The MIM complex is proposed to have a major role in this pathway; it can mediate the transfer of substrates from the TOM complex as well as promote the import and assembly of all types of α-helical proteins (Table 2).^2,123^

**Table 2:** Targeting and sorting signals for the import of mitochondrial proteins

Proteins targeted to:	Targeting signal	Description	Import machinery	Example
Matrix	N-terminal cleavable presequence	• Amphipathic α-helix	• Receptors: Tom20, Tom22, Tom70	TRX3: thioredoxin required to maintain redox homeostasis in the cell
		• 15–60 residues	• Translocases: Tom40, Tim23	
		• Net charge of +3 to +6		
		• High content of hydroxylated residues		
		• MPP cleavage site		
Inner membrane	Internal targeting signal	• Six α-helical transmembrane domains	• Receptors: Tom70	OXA1: mitochondrial inner membrane insertase
			• Translocases: Tom40, Tim22	
Outer membrane	Signal-anchored	• N-terminal α-helical transmembrane domain	• Receptors: Tom70	Tom70: component of the TOM complex; acts as a receptor for incoming precursor proteins
			• Translocases: Tom40, MIM complex	
	Tail-anchored	• C-terminal α-helical transmembrane domain	• Receptors: Tom70	Tom5: component of the TOM complex; involved in transfer of precursors from the Tom70 and Tom20
			• Translocases: Tom40, MIM complex	
	Multiple transmembrane domains	• Multiple α-helical transmembrane domains	• Receptors: Tom70	Tom22: component of the TOM complex; mediates interaction between TOM and TIM complexes
			• Translocases: Tom40, MIM complex	
	β-Barrels	• Multiple transmembrane β-strands	• Receptors: Tom20	Tom40: component of the TOM complex; constitutes the core element of the protein conducting pore
		• Most C-terminal β-strand is the targeting signal (β-signal)	• Translocases: Tom40, SAM complex	
IMS	N-terminal cleavable presequence with stop-transfer sequence	• Amphipathic α-helix	• Receptors: Tom20, Tom22	GUT2: mitochondrial glycerol-3-phosphate dehydrogenase
		• 15–60 residues	• Translocases: Tom40, Tim23	
		• Hydrophobic sorting region		
		• IMP cleavage site		
	Internal targeting signal with characteristic Cys motif	• Amphipathic α-helix	• Receptors: Tom20	Erv1: flavin-linked sulfhydryl oxidase of the mitochondrial IMS, oxidizes Mia40 as part of the disulfide relay system
		• 9 amino acids long	• Translocases: Tom40, Mia40	
		• Hydrophobic residues in position −3 and −4		
		• Aromatic residue in position −7		

## 4. Chaperone-assisted protein folding in the IMS

The specific translocon machineries and their interaction with the variety of targeting signals that we have discussed so far ensure the correct targeting and sorting of the imported proteins to the different mitochondrial sub-compartments. However, as the transported proteins are targeted to the organelle in a largely unfolded state (which allows them to thread efficiently through the narrow translocon channels), they need to be folded at the *trans* site inside the organelle after the transport process has been completed. The newly synthesised proteins that are to be transported to mitochondria are kept unfolded by a system of cytosolic chaperones to avoid protein aggregation and premature damage.^126^ In mitochondria, dedicated molecular chaperones assist the folding of newly imported proteins and prevent their aggregation while their hydrophobic segments are transported through the aqueous sub-compartments.

In the mitochondrial matrix, various molecular chaperones have been identified that help newly imported proteins to fold. These belong to the well characterised families of Hsp70 Hsp60 and their cochaperones Hsp40 an Hsp10. One key chaperone in the matrix is for example the mitochondrial heat shock protein (mtHsp70) is an ATP-dependent import motor of the TIM23 complex that not only helps drive protein translocation, but also mediates the folding of the imported substrates.^24,127^

By contrast, the IMS presents several unique challenges as a folding environment for the newly import proteins. First, the IMS does not retain its own pool of ATP as the matrix, and in fact there are no ATP-dependent chaperones in the IMS.^128^ Second, the IMS is the most constricted mitochondrial sub-compartment, with local concentrations of proteins very high and an increasing potential for aggregation, particularly for proteins with exposed hydrophobic patches which are common for the unfolded imported proteins. Finally, the extended physical interactions between the outer and inner membranes in contact sites and the internal segregation of the cristae lumen separately from the boundary intermembrane space put further constraints on the folding process in this compartment.

In this context, we will discuss in this chapter the three main chaperone systems in the IMS. Namely, we will focus on the structure and chaperone function of Mia40 as a holdase, the function of the small Tims as dedicated membrane protein chaperones and finally the chaperone role of the protease Yme1.

### 4.1 The Mia40 holdase

**Fig. 5:**
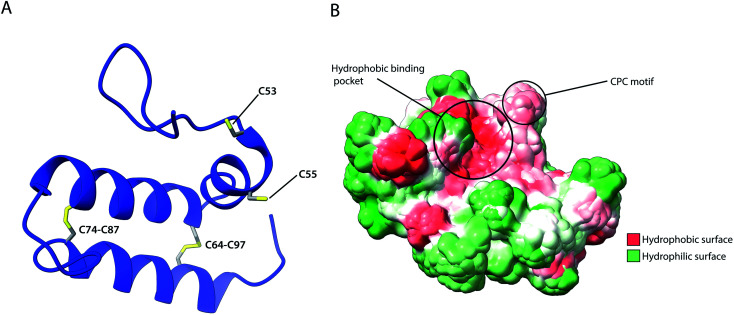
Structural features of human Mia40. (A) Structure of the protein core (residues 49–109), highlighting intramolecular disulphide bridges as well as Cys53 and Cys55 of the catalytically active CPC motif. (B) Hydrophobicity surface, highlighting the hydrophobic substrate binding cleft and the adjacent CPC motif (PDB ID: 2K3J).

As previously described, several IMS proteins are required to go through oxidative folding during maturation by interaction with an oxidoreductase, namely Mia40 in yeast or its human homolog CHCHD4.^53,56^ The most notable difference between the two homologs is that the yeast variant contains a large N-terminal extension of 242 amino acids that tethers the protein into the inner membrane facing the IMS, while this segment is absent in CHCHD4 resulting in the protein being entirely soluble in the IMS. Despite their variance, they share a high sequence identity in the central and catalytically active region of the protein (residues 47–107 in CHCHD4 and residues 290–350 in yeast) which include the conserved cysteine residues in a –CPC–CX_9_C–CX_9_C– motif. The Nuclear Magnetic Resonance (NMR) structure of this segment for the human protein (Fig. 5) revealed the protein core to be composed of 3 helices; α1 (residues 56–59) which is a very short helix that contains the redox-active CPC motif and the only defined secondary structure of the N-terminal segment of the protein core; α2 (residues 65–77) and α3 (residues 88–100) form an antiparallel α-hairpin that is stabilized by the disulphide pairs Cys64–Cys97 and Cys74–Cys87. The NMR data obtained also suggests that the N-terminal segment varies in flexibility upon disulphide bond formation in the CPC motif, thus otherwise referred to as the “lid”.^53^ Mia40 substrates share a coil–helix coiled coil–helix (CHCH) motif that is imported into mitochondria in a reduced and unfolded form.^34,52^ The oxidative folding ability of Mia40 depends on the formation of transient intramolecular disulphide bridges between the Cys55 of the CPC motif and specific cysteines of the substrate proteins. Successful binding achieves a rapid disulphide exchange that results in the CHCH domain of substrates being oxidised while Mia40 is reduced. Such exchanges include the Cys45 in the CX_9_C motif of Cox17,^53^ and the first cysteine of the CX_3_C motif in Tim9 and Tim10 that regulates the assembly of the TIM9·10 complex.^37,129^ Aside from the oxidative function of Mia40, the orientation of the substrate with the correct Cys to the CPC motif was found to be crucial to achieving efficient disulphide exchange and strictly dependent on hydrophobic packing between the ITS and the Mia40 substrate cleft.^57^ The Mia40 structure revealed several conserved hydrophobic residues on the α-helix opposite the CPC motif made up of residues Leu42, Ile43, Ile49, Trp51, Leu56, Met59, Ala60, Phe68, Phe72, Phe75, Phe91, Met94 and Met98. These residues were collectively shown to be essential for cell survival.^53^ The hydrophobic collapse of the ITS hydrophobic residues, that make up the solvent-exposed hydrophobic binding cleft of Mia40, stabilise the unfolded substrate in the IMS milieu, with Mia40 functioning as a holdase.^57,130^ This initial step of substrate binding to Mia40 (and hence its chaperone activity) is independent of subsequent oxidation (*i.e.* disulphide bonding) steps.^57,112^ In fact, the hydrophobic interactions dictated by the substrate binding cleft of Mia40 are also involved in the recycling of Mia40 by Erv1. In this reaction, Erv1 binds to Mia40 in a manner that mimics the substrate (‘substrate mimicry model’) and making use of an extended hydrophobic patch in the intrinsically disordered N-terminal of Erv1.^62,63^ While Mia40 catalyses disulphide bridges that thermodynamically favour α-helical folding for its substrates,^131^*in vitro* experiments of Koch *et al.*, with Mia40 and Cox17 proposed an additional proofreading ability for the chaperone by re-shuffling incorrect disulphides within its substrate protein.^132^

### 4.2 The small Tim chaperones for polytopic proteins

**Fig. 6:**
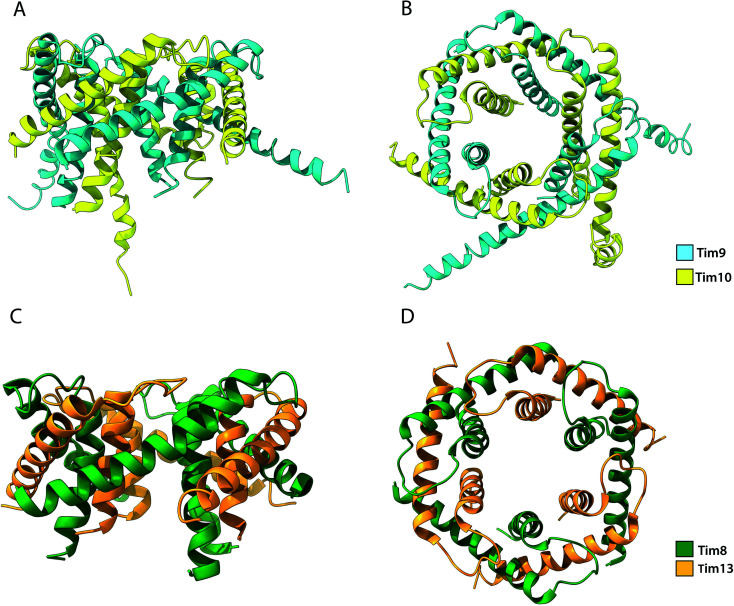
Structure of the Tim9·10 and Tim8·13 complex. (A) Side view and (B) top view of the Tim9·10 complex (PBD ID: 2BSK). (C) Side view and (D) top view of the Tim8·13 complex (PDB ID: 3CJH).

Among the substrates of Mia40 are the small Tims, a family of ∼10 kDa chaperones that exist within the IMS of mitochondria. Their most well studied function is the transport metabolite carriers across the IMS, by protecting exposed hydrophobic regions of these integral membrane proteins.^35,36,108^ Additionally, the small Tims also assist the integration of β-barrel protein in the OM of mitochondria, making them the only known chaperone system capable of interacting with both types of membrane proteins (β-barrels and α-helical) the family members include Tim8, Tim9, Tim10, Tim12 and Tim13 in *S. cerevisiae* and are organised in specific oligomeric assemblies. Tim9 pairs with Tim10 and Tim8 with Tim13. They share a conserved CX_3_CX_*n*_CX_3_C motif (where *n* ∼ 15) or two CX_3_C motifs that are essential for their function.^52,129^ Tim9 and Tim10 are essential proteins^35,133^ that share 22% sequence identity and 50% sequence similarity. By contrast, Tim8 and Tim13 are not essential for viability. Following their import into the IMS, they assemble into a thermodynamically stable 70 kDa hexameric complex.^37,134^ Once assembled, Tim12, an essential subunit of the TIM22 complex binds the Tim9·10 complex to enable (i) substrate insertion into the inner membrane and (ii) dual localisation for the chaperone, in the bulk of the IMS and tethered to the inner membrane in a Tim9·10·12 complex intermediate.^35,135^ Folding of the unassembled Tim9 and Tim10 requires their oxidation by Mia40 and it is only the oxidised and fully folded Tim9 and Tim10 that can assemble to form a functional heterohexameric Tim9·10 chaperone complex.^52,129,134^ The secondary structure of the complex is exclusively α-helical, where the two subunits alternate in a 6-fold molecular symmetry to form a closed circular core. Each subunit folds into a helix-loop-helix as disulphide bridges in-between CX_3_C motifs create a central loop. The whole complex comprises 6 inner and 6 outer helices (Fig. 6). In the central part of the subunit, they form a doughnut-shaped substrate-binding core around a hollow pore of 15 Å, while the antiparallel N- and C-termini extend downward of the core.^134^ Depending on the length of the transferred protein, multiple chaperones may attach to the same chain for its efficient protection.^136^ Sequence analysis of small Tims identified a set of conserved hydrophobic residues that face the binding cleft found in-between the inner and outer helices in both Tim9·10 and Tim8·13 complexes.^137^ Mutagenesis of these conserved residues to hydrophilic residues in Tim9·10 resulted in cell death and are thus proposed to be essential for the biogenesis of its substrate proteins.^136^ However, it could be argued that replacing multiple residues in the protein core with amino acids of inverse physicochemical properties likely affects the structure of the complex and elicit this cell response. The ADP/ATP carrier protein AAC has been widely reported to bind the Tim9·10 complex during its transport through the IMS to the translocase Tim22.^36,105,134^ While the transmembrane domains of AAC bind the Tim9·10 complex core,^36^ Tim10 was shown to interact with AAC using its N-terminal domain. The individual subunits' role in substrate binding suggests that Tim9 has more of a structural role within the complex while Tim10 maintains substrate specificity and affinity.^105^ Though not essential in yeast, the Tim8·13 complex was shown to be most important in low membrane potential conditions.^39^ Import of its primary substrate, Tim23 is heavily dependent on membrane potential. By binding the N-terminal domain of Tim23, the Tim8·13 complex prevents protein aggregation during import as well as prevents its retrograde translocation.^39,41^ The Tim8·13 complex shares structural similarities with Tim9·10 but have been shown to have different substrate-binding preferences, as well as additional hydrophilic interactions not reported in the Tim9·10 complex. It appears the two complexes have separate roles within the IMS. However, membrane precursor proteins were shown to be transferrable from one TIM chaperone to the other, indicating a possibility for cooperation in their chaperone activity.^137^

### 4.3 The Yme1: a protease with a chaperone activity

Yme1 is a member of conserved family of AAA+ ATPases that are associated with a plethora of cellular processes.^138^ These include: cellular quality control, organelle biogenesis, membrane fission and vesicular transport.^139^ Failure to sustain protein quality control may lead to impaired cellular function and as a result the development of numerous human diseases.^140^ Yme1 is involved in mitochondrial protein quality control and has been specifically associated with the degradation or unfolded or damaged proteins. However, further research has revealed an additional role of Yme1 as a molecular chaperone assisting the folding of newly imported polypeptides.^141,142^

**Fig. 7:**
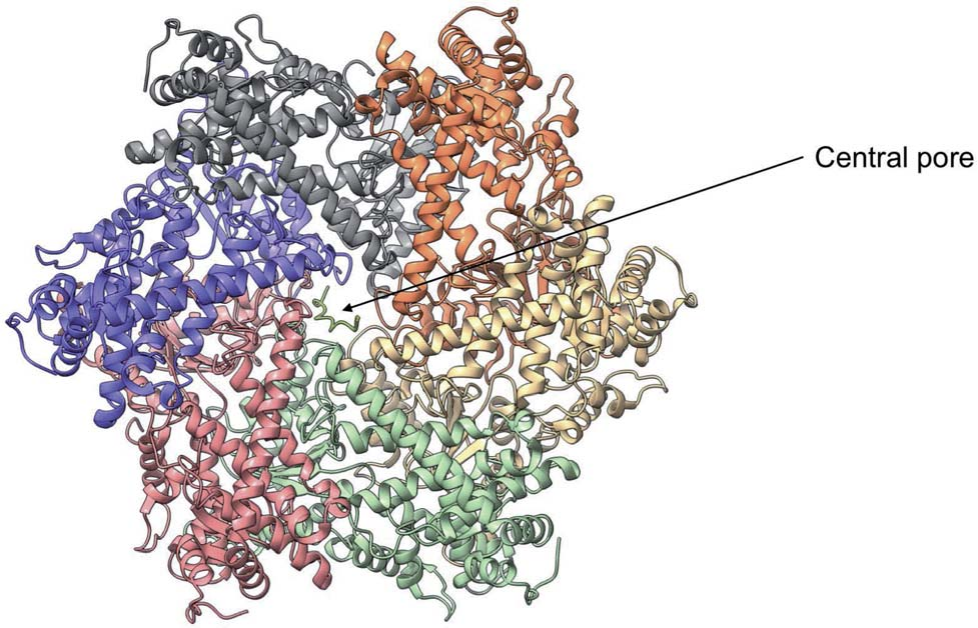
Cryo-EM structure of Yme1. This is the top view of the protein showing the C6-symmetric protease ring that faces the intermembrane space. A central pore of ∼1.4 nm diameter is formed where the substrate binds (PDB ID: 6AZ0).

**Fig. 8:**
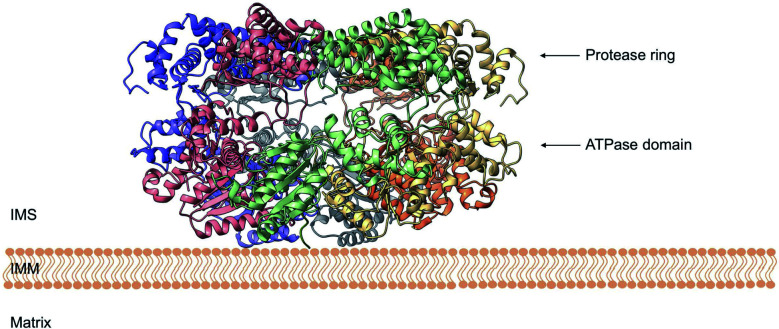
Side view of the cryo-EM structure of Yme1. The protein is tethered to the inner mitochondrial membrane (IMM) by a membrane helix. The subunits of the ATPase domain create an asymmetric staircase while the protease ring subunits create a planar symmetric C6 ring. The purple and green structures represent the different translocases found on the IMM (PDB ID: 6AZ0).

Despite the crucial function of Yme1, little was known about is structure until Puchades *et al.*, took on the arduous task of resolving its cryo-EM structure.^138^ Yme1 is a hexameric protein and every subunit contains an ATPase and peptidase domain (Fig. 7). As seen in Fig. 8, Yme1 is tethered to the inner membrane by a membrane helix and its catalytic domains are facing the IMS.^143^ Yme1 assembles into two stacked rings, with the ATPase domains creating an asymmetric spiral staircase above the protease ring. The staircase creates a central pore (∼1.4 nm diameter) with a conserved aromatic-hydrophobic motif that is essential for binding substrate proteins. ATPases are ATP-driven machineries that are characterised by a conserved P-looped AAA domain containing Walker A and Walker B motifs. The AAA domain of Yme1 contains a conserved Walker A motif, with the pattern G-X(4)-GK-[TS].^144^ Numerous studies show the involvement of the motif in recognising and binding substrates for translocation or degradation. It is also hypothesised that this motif is involved when Yme1 binds substrates to prevent aggregation or mediate protein folding.^141^

The AAA domain of Yme1 was first shown to act as a chaperone by binding to unfolded protein dihydrofolate reductase (DHFR) in *in vitro* experiments using isolated mitochondria.^141,145^ Moreover, it was suggested that Yme1 assists with the folding/assembly of Cox2, a subunit part of the catalytic core of cytochrome c oxidase. Thus, supporting that Yme1 acts as a chaperone also *in vivo* conditions.^146^ Later research confirmed the role of Yme1 as a folding helper for DHFR in the IMS. Importantly, the chaperone activity of Yme1 was independent from its protein degradation function. The study also identified numerous endogenous substrates that aggregate in the absence of Yme1. These proteins are necessary for the aforementioned functions of the IMS, thus further supporting the importance of Yme1.^142^ Interestingly, Erv1 was identified as one of the endogenous substrates of Yme1. As mentioned above, Erv1 is an essential component of the oxidative folding pathway. Therefore, Yme1 presumably has a crucial role in a variety of mitochondrial pathways.

Despite the growing evidence in support of Yme1 as a chaperone in the IMS, the mechanism of its function is not yet clear. It is hypothesized that Yme1 binds newly imported proteins during their translocation and assists with their folding. Another hypothesis is that the AAA domain of Yme1 binds unfolded proteins and helps keep them in a folding-competent state to enable their refolding. If folding is unsuccessful, then the proteins get degraded by the proteolytic domain of Yme1.^142^ The cryo-EM structure of Yme1 has given a better picture of the architecture of the protein and the residues involved in substrate binding.^138^ Nevertheless, the exact mechanism of the chaperone activity of Yme1 still needs to be determined.

The different chaperone systems in the IMS (Mia40, small Tims and Yme1) seem to display specificity against their substrates with no overlap in their function. However, the small Tims interact both with Mia40 as a substrate and they also seem to be subject to quality control by the Yme1. It is not yet very clear whether other proteins may be processed by more than one of the IMS chaperone systems.

## 5. Conclusions

Mitochondria have developed multiple import pathways to accommodate the import of the different types of proteins needed for mitochondrial function. Most mitochondrial sub-compartments have typically one or two major import pathways, but the intermembrane space seems to divert with a very high variety of pathways, some of which are dictated by targeting signals that are still either completely unknown or poorly understood. This is surprising given that the IMS is the smallest mitochondrial sub-compartment, but it is perhaps indicative of the pleiotropic function of the IMS.

The emergence of new IMS proteins carrying atypical signals is of great interest as not much is known about targeting signal specificity and its recognition by the various import components. Furthermore, there is growing evidence that proteins like the Mia40, the small Tims and Yme1 have more functions than previously anticipated. Taken together, these might suggest that mitochondria have found ways to evolve in order to recognise novel targeting signals or have developed such alternative import pathways as a backup when mitochondrial function is impaired. Thus, it is important to dissect how these unconventional targeting pathways work in order to better understand mitochondrial function and how it can be altered to prevent disease progression.

## Conflicts of interest

The authors declare no conflict of interests.

## Supplementary Material
